# The research landscape and evolutionary trends of platelet-derived extracellular vesicles: a bibliometric and LDA analysis (2015–2026)

**DOI:** 10.3389/fonc.2026.1854626

**Published:** 2026-07-08

**Authors:** Liuxin Yu, Xiaoping Yan, Hongyu Wang, Hongwei Zhang

**Affiliations:** 1 Class 2024, School of Basic Medical Sciences, Southwest Medical University, Luzhou, Sichuan, China; 2 Department of Blood Transfusion, The Affiliated Hospital of Southwest Medical University, Luzhou, Sichuan, China; 3 Department of Blood Transfusion, Zigong Third People’s Hospital, Zigong, Sichuan, China

**Keywords:** bibliometrics, extracellular vesicles, oncology, platelets, regenerative medicine

## Abstract

**Introduction:**

Since the 2013 Nobel Prize in Physiology or Medicine was awarded, extracellular vesicles(EVs) have made the transition from the laboratory to clinical practice. Platelets, as emerging multifunctional immune cells, have garnered significant attention due to the natural biocompatibility and low immunogenicity of platelet-derived extracellular vesicles (pEVs). However, research in this field currently lacks comprehensive review and analysis.

**Methods:**

This study utilized the Web of Science database, along with tools such as VOSviewer and CiteSpace, and employed bibliometric analysis and Latent Dirichlet Allocation (LDA) to systematically examine international collaboration, thematic evolution, and research hotspots in this field. In addition, clinical trials related to pEVs were extracted from the PubMed database to assess translational progress.

**Results:**

Since 2021, the volume of publications in the field of pEVs has remained stable at a high level. The International Journal of Molecular Sciences was the most productive journal. The United States and China were the leading contributing countries, with the United States occupying a central position in global collaboration networks. Harvard Medical School and Eric Boilard were among the most influential institution and author, respectively. Research hotspots have shifted from basic platelet biology and cardiovascular studies toward regenerative medicine and oncology. Clinical trials remain limited and primarily focus on pathophysiological mechanisms, biomarker applications, and therapeutic exploration.

**Discussion:**

This study provides a comprehensive visualization of the global research landscape and developmental trends in pEVs from 2015 to 2026. Although basic research has expanded rapidly, clinical translation remains limited, indicating a clear gap between laboratory findings and clinical application. Emerging trends highlight increasing attention toward regenerative medicine, oncology, and advanced therapeutic strategies, providing directions for future research.

## 1 Introduction

Platelets have traditionally been recognized for their indispensable roles in hemostasis and thrombosis. However, accumulating evidence has demonstrated that platelets also actively participate in a wide range of physiological and pathological processes, including inflammation, immune regulation, angiogenesis, tissue regeneration, and tumor progression (1). Owing to these diverse biological functions, platelets have attracted increasing attention not only as key regulators of disease pathogenesis but also as promising biomarkers, therapeutic targets, and biological delivery vehicles in translational medicine. EVs are lipid bilayer-delimited particles released from cells that serve as important mediators of intercellular communication through the transfer of proteins, lipids, nucleic acids, and other bioactive molecules (2). Platelets constitutively release EVs under physiological conditions, whereas platelet activation, apoptosis, and ex vivo storage may substantially influence EV release, composition, and biological activity (3). Rather than being equivalent to their parent cells, pEVs retain selected molecular and functional characteristics inherited from platelets while also possessing distinct biological properties. In terms of surface molecular characteristics, pEVs retain various membrane proteins and adhesion molecules from platelets, including typical platelet markers such as integrin αIIb (CD41), glycoprotein Ibα (GPIbα/CD42b), integrin β3 (CD61), and P-selectin (CD62P) (4). With respect to molecular cargo, pEVs contain numerous platelet-derived proteins, nucleic acids, and lipid components, including growth factors, microRNAs (miRNAs), and mitochondria-related constituents (5). Existing evidence suggests that pEVs may recapitulate certain platelet-associated biological functions, including procoagulant and immunomodulatory activities, under specific physiological and pathological conditions (6). At the same time, pEVs serve as important mediators of intercellular communication and contribute to diverse physiological and pathological processes, including coagulation, inflammation, and tissue repair.

Between 2014 and 2023, the International Society for Extracellular Vesicles (ISEV) successively released the Minimal Information for Studies of Extracellular Vesicles (MISEV) 2014, 2018, and 2023 guidelines, which have progressively refined recommendations regarding the definition, nomenclature, classification, characterization, and reporting of EVs (7–9). MISEV2023 recommends that operational terms used as prefixes for “EVs” should be applied with caution when EV subtypes are distinguished based on characteristics such as size, density, molecular composition, or cellular origin. Nevertheless, the pEV field continues to employ a variety of terms, including “exosomes, “ “microvesicles, “ “ectosomes, “ “apoptotic bodies, “ “small extracellular vesicles (sEVs, typically <200 nm), “ and “large extracellular vesicles (lEVs, typically >200 nm).” Earlier studies also used historical terms such as “nano-vesicles”. However, because these terms lack clear biological definitions, they are now rarely adopted as standardized EV classification nomenclature. In recent years, substantial progress has been made in pEV research. However, to date, only one bibliometric study has systematically reviewed this field, and its analysis focused specifically on platelet exosomes (referred to as “platelet exosomes” in the original study), which represent only one subtype of EVs. Therefore, to remain consistent with the majority of the published literature and to provide a more comprehensive bibliometric overview of the field, the term “platelet-derived extracellular vesicles” (pEVs) was used throughout the present study.

As a tool that employs mathematical and statistical methods to analyze the distribution patterns and intrinsic relationships of scholarly literature, bibliometrics clearly reveals the core players, hot topics, and developmental trajectories within a research field, providing researchers with valuable guidance for understanding field dynamics and determining research directions (10). Therefore, this study draws on the researchers’ in-depth understanding of EVs (6, 11)and combines bibliometric methods with LDA topic modeling to systematically analyze literature on pEVs published between January 1, 2015, and February 28, 2026, thereby constructing a rigorous and comprehensive research landscape. The objective of this study is to use visual analysis to map the current state of research in this field, elucidate collaboration networks and emerging trends, depict the temporal evolution of research themes, and summarize progress in clinical research, thereby providing rigorous and actionable guidance for subsequent mechanistic studies and clinical trial planning.

## 2 Materials and methods

### 2.1 Data sources and search strategy

**Figure 1:**
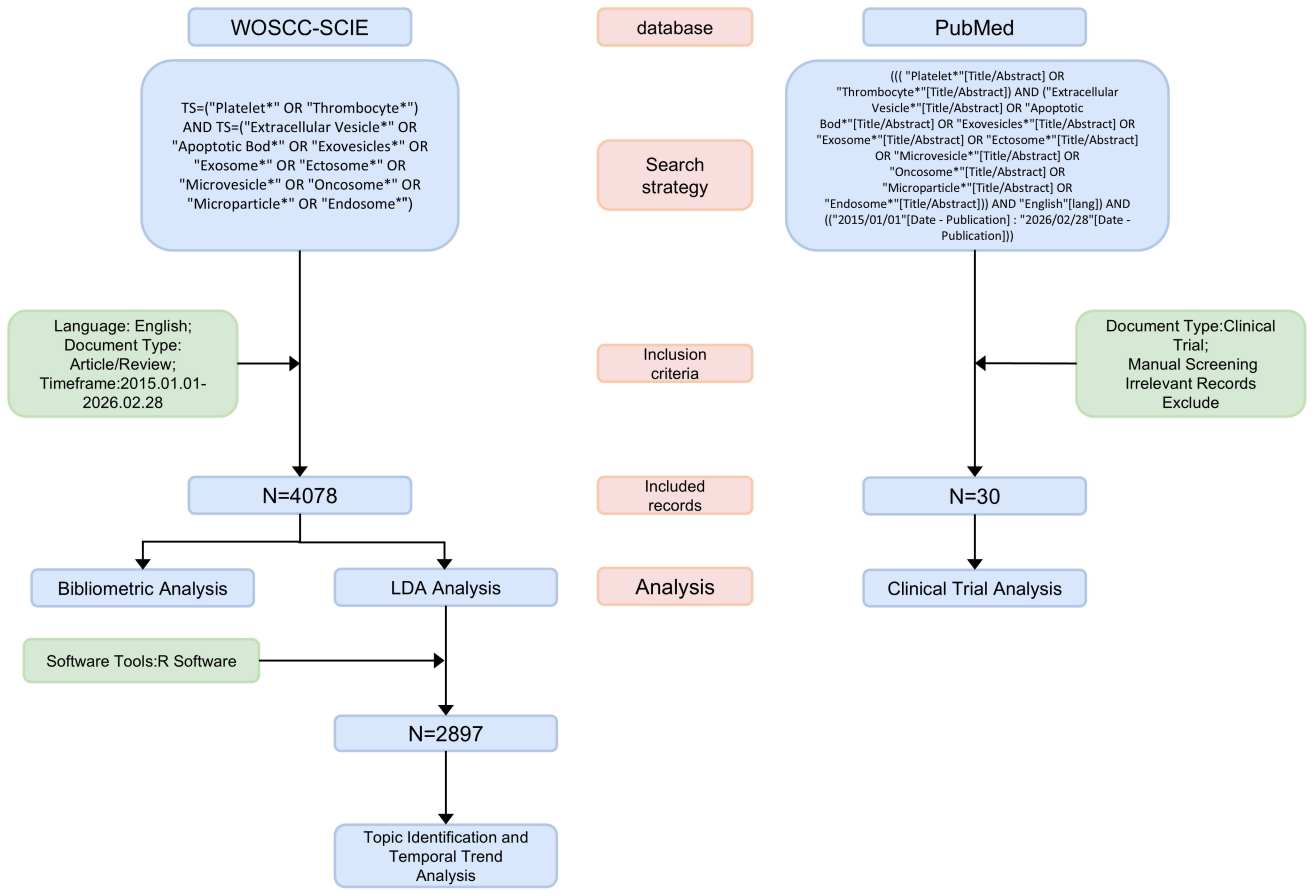
Flowchart of study selection and data processing for bibliometric and LDA analyses. For the Web of Science Core Collection Science Citation Index Expanded (WOSCC-SCIE), records were initially screened according to the inclusion criteria. A total of 4, 078 records were included for bibliometric analysis and latent Dirichlet allocation (LDA)-based topic modeling using R software. After additional quality control and data preprocessing, 2, 897 records were retained for topic identification and temporal trend analysis. For PubMed, only publications classified as clinical trials were included. After manual screening to exclude irrelevant studies, 30 clinical trials were ultimately retained for clinical trial analysis.

To investigate research trends and clinical translation in the field of pEVs, this study utilized two authoritative databases: the Web of Science Core Collection (WoSCC) and PubMed. These two databases have distinct roles and complement each other. The primary data used in this study were retrieved from the Science Citation Index Expanded (SCI-EXPANDED) within the Web of Science Core Collection on February 28, 2026, using the following search strategy: (TS=(“Platelet*” OR “Thrombocyte*”) AND TS=(“Extracellular Vesicle*” OR “Apoptotic Bod*” OR “Exovesicles*” OR “Exosome*” OR “Ectosome*” OR “Microvesicle*” OR “Oncosome*” OR “Microparticle*” OR “Endosome*”)). The document type was restricted to “article” or “review, “ the language was set to English, and the publication date range was set from January 1, 2015, to February 28, 2026. In the WoSCC database, the document type “Article” was used according to the database classification system and includes original research articles, basic research articles, and other primary research reports indexed as Articles by WoSCC. The search results were exported as a plain text file in the “Full-text and Cited References” format. After deduplication using Citespace, 4, 078 articles were identified for subsequent visualization. The clinical trial search results obtained from the PubMed database were exported in PubMed format; and after excluding irrelevant studies, the final 30 clinical trials were used to summarize the progress of clinical research. For details on the specific search strategy and inclusion criteria, please refer to the appendix. The literature screening process is shown in **Figure 1**.

### 2.2 Bibliometric analysis

Bibliometric analyses were performed using multiple software packages and online visualization platforms. Microsoft Excel was used to analyze annual publication output, publication trends, and author productivity. Collaborative relationships among authors, countries, and keywords were visualized using R packages, while Scimago Graphica was employed to map international research collaborations. Institutional collaboration networks, journal co-citation networks, and temporal and density visualizations were constructed using VOSviewer. In addition, CiteSpace was applied to perform keyword burst detection, thereby identifying emerging research hotspots and developmental trends in the field. Journal and institutional publication outputs were further visualized using the online platform Flourish Studio (https://flourish.studio/).

### 2.3 Topic modeling

To uncover the thematic structure within the corpus, we employed the LDA model. This model is a hierarchical Bayesian extension of probabilistic latent semantic analysis (PLSA) and is capable of effectively identifying latent themes in large-scale text data (12). In this study, 4, 078 deduplicated articles were used for visualization analyses such as collaboration networks and keyword co-occurrence. Because text mining places high demands on data integrity and validity, we used R software to preprocess the 4, 078 articles, ultimately including 2, 897 valid articles. We utilized the quanteda package for text preprocessing. After constructing the corpus, the text was uniformly converted to lowercase, and punctuation, numbers, symbols, and delimiters were removed. Common English stop words and meaningless high-frequency words were removed, while core terms such as “platelet” and “extracellular vesicles” were retained. Blank tokens and single-character meaningless tokens were removed, and documents with no valid content were filtered out. The final corpus consisted of 2, 897 articles and reviews.

We generated a token set combining words and binary syntax, and constructed a document-term matrix (DTM), retaining terms that appeared in at least three documents while removing high-frequency generic words that appeared in more than 90% of the documents. Finally, we used the `topicmodels` package to construct an LDA topic model and trained the model using Gibbs sampling. By combining confusion matrix analysis with topic consistency analysis, we ultimately determined the optimal number of topics to be k=8, with α=50/k, β=0.01, and 2, 000 iterations. To further evaluate the model’s stability and effectiveness, we conducted validation from three perspectives. First, we calculated the model’s confusion index based on the training dataset. Second, we assessed topic stability through 10 bootstrap resamples and used Jaccard similarity to calculate the match between the top 10 core terms of each topic in the original model and the resampled models. Finally, we quantified the semantic coherence and domain relevance of the topics based on the co-occurrence frequency of terms in the literature. We manually review the top 20 high-weight terms for each topic, assign interpretable topic labels to each topic, and build a linear regression model for each topic to illustrate its temporal trends.

## 3 Results

### 3.1 Growth trends and overall publication dynamics

**Figure 2:**
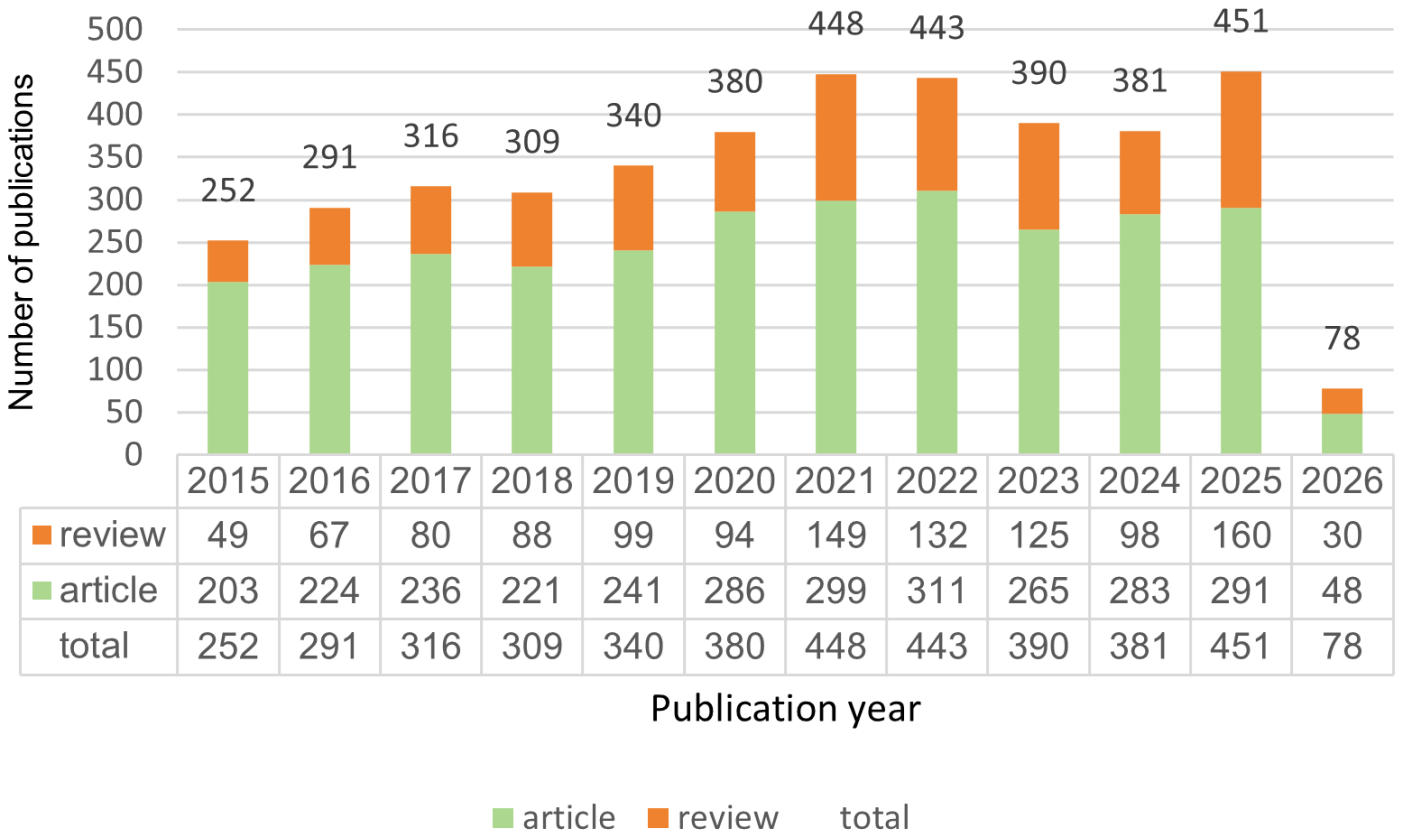
Annual publication output on pEVs research from 2015 to 2026. The stacked bar chart illustrates the yearly number of publications indexed in WoSCC, categorized into original research articles (green) and review articles (orange). The total number of publications for each year is shown above the bars.

Analysis of publication trends can provide valuable insights into the developmental trajectory of a research field, thereby helping researchers evaluate its research momentum and guide future research planning. A total of 4, 078 publications were included in the analysis. From 2015 to 2025, the number of publications increased from 252 to 451, representing an overall growth of approximately 79%. During the early phase of the research (2015–2020), publication output increased steadily from 252 to 380 articles, with original research articles accounting for the majority of publications. In 2021, the annual publication volume exceeded 400 for the first time, suggesting a further expansion of research activity in this field. Between 2023 and 2025, although annual publication output showed minor fluctuations, the total number of publications reached its highest level in 2025. Among these, the number of articles remained relatively stable, while the number of reviews showed fluctuating growth in the later period and accounted for approximately 35% of publications in 2025, reflecting the progressive maturation of the field. As the search was limited to February 28, 2026, only 78 publications have been identified for 2026 so far. (**Figure 2**).

### 3.2 Global distribution and international collaboration patterns

**Figure 3:**
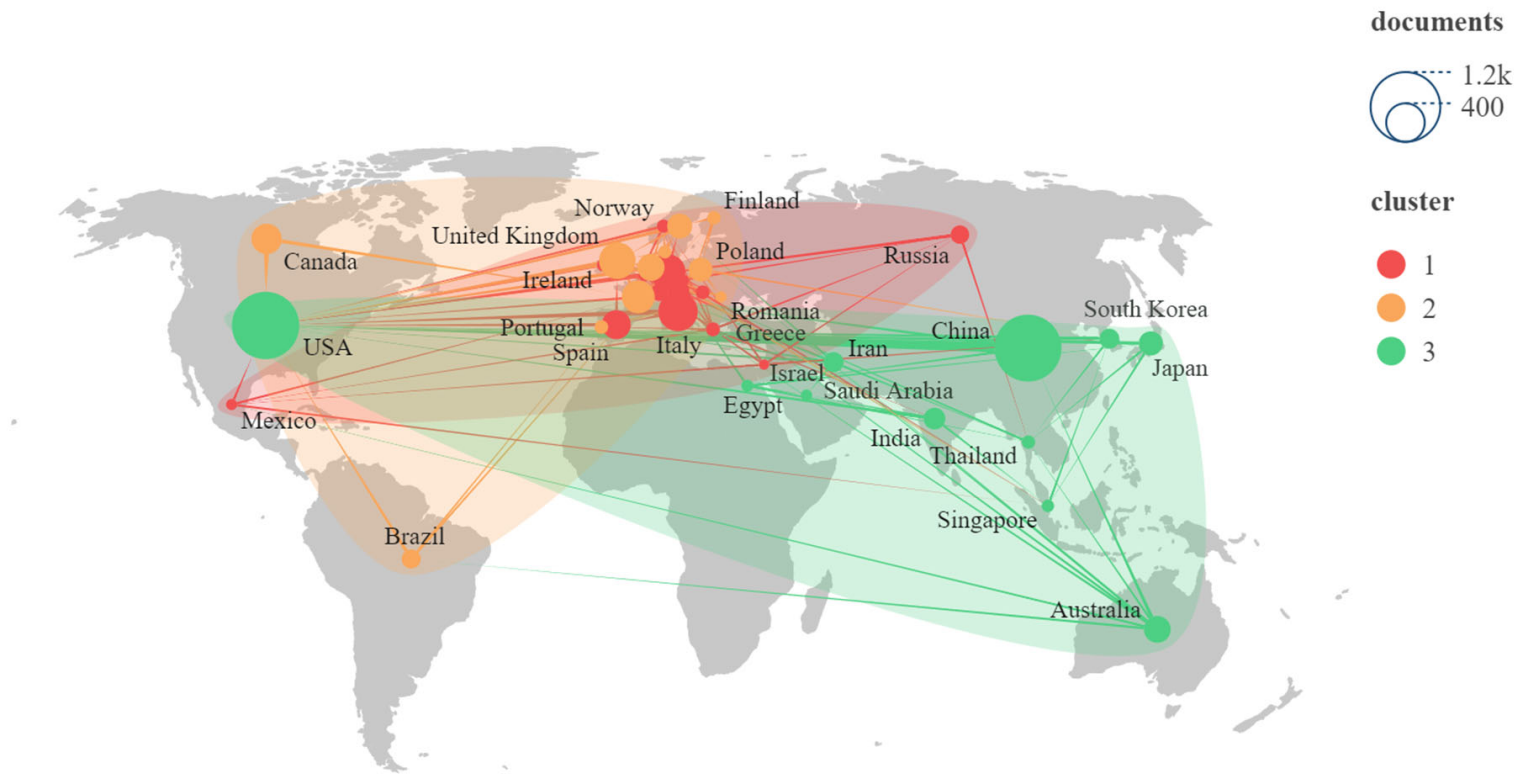
World map of international collaborations in pEVs research. The map visualizes the global distribution of collaborative research activities among countries/regions involved in pEVs-related publications. Different colors represent distinct collaboration clusters identified through bibliometric network analysis, reflecting groups of countries with relatively stronger internal collaborative relationships. Three major collaboration clusters were identified, centered primarily on Italy, the United States, and China, respectively. Connecting lines indicate cooperative relationships between countries/regions, with thicker lines representing stronger collaboration intensity, whereas node size corresponds to the publication output of each country/region.

From the perspective of national research output and collaboration networks, bibliometric analysis can reveal the major driving forces and collaborative patterns of global research, thereby providing valuable references for researchers and policymakers seeking to strengthen international scientific cooperation. A total of 93 countries participated in research on pEVs. The international collaboration network is presented in **Figure 3**. The United States occupied a central position in global collaboration, with the highest number of publications (n=997) and a total link strength of 713, indicating extensive international collaboration and influence within this research field. China and the United States demonstrated the strongest collaboration (link strength = 139), suggesting that they currently represent major contributors to global pEV research.

In addition, countries such as South Korea, Germany, and the United Kingdom exhibited relatively high node centrality, indicating important bridging roles in international collaboration. International collaboration in this field has gradually formed relatively stable regional cooperative patterns, mainly consisting of three major research clusters.

To assess whether high-output countries introduced bias into research hotspots and their temporal changes, two sensitivity analyses were performed. In the leave-one-country-out analysis, most core keywords (occurring ≥60 times) maintained Pearson correlation coefficients ≥0.8 and absolute differences in correlation coefficients ≤0.1 after excluding China or the United States, indicating high overall consistency. For some keywords (“ANGIOGENESIS” and “EXOSOME”), consistency decreased when China was excluded, which may be attributed to their sparsity and regional concentration. Balanced Bootstrap analysis further validated the reliability of the keyword frequency analysis in this study. Results from 500 resampling runs addressing the disparity in the number of Chinese and American publications showed that the relative error between the Bootstrap-averaged relative frequencies and the original population frequencies for the 28 core keywords was <5% in all cases, with narrow 95% confidence intervals. The two methods provided complementary validation, confirming that the annual evolutionary trends and frequency distribution characteristics of the research topics are not affected by the heterogeneity of literature from high-output countries, demonstrating strong robustness.

### 3.3 Leading institutions and collaborative networks

**Figure 4:**
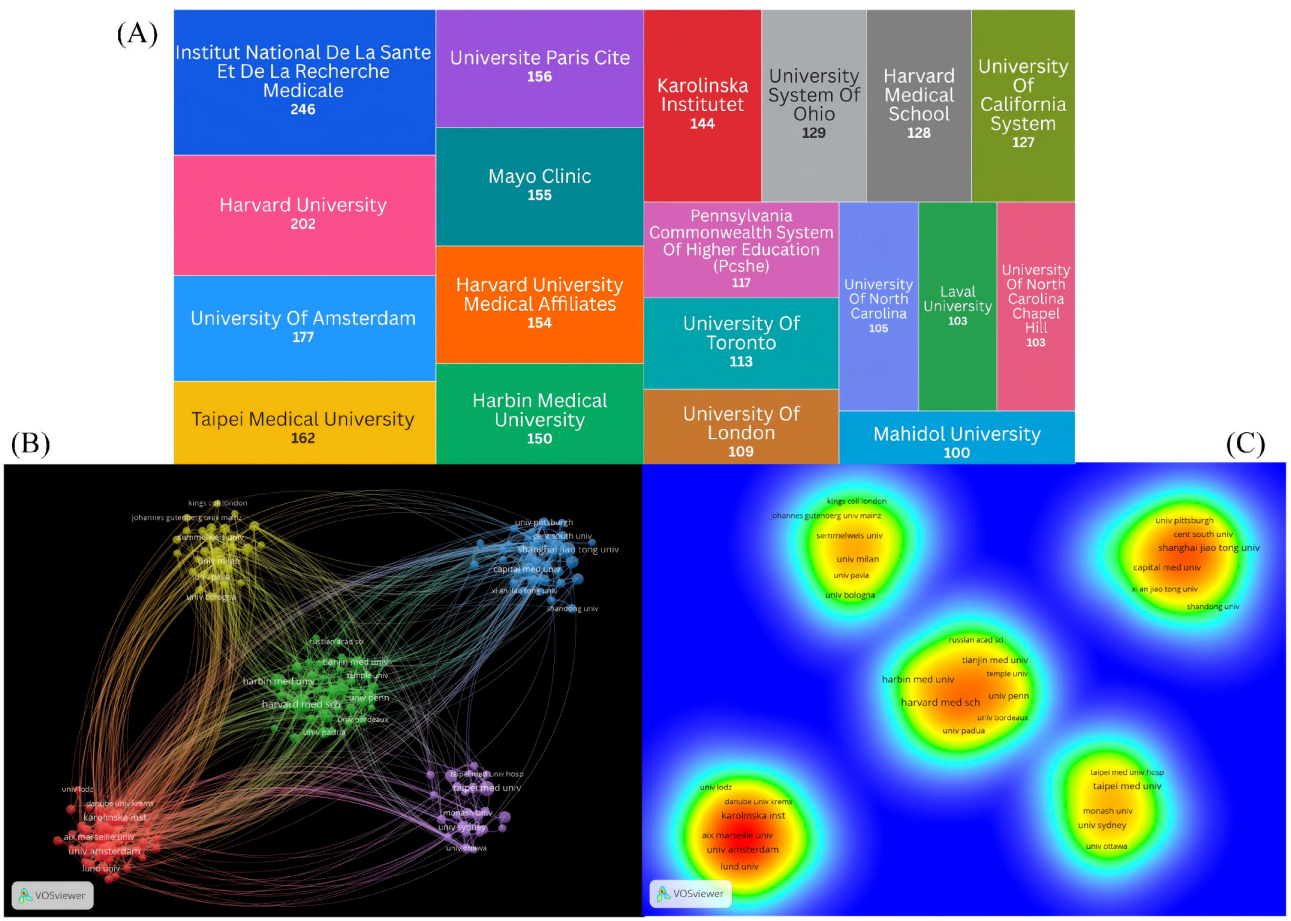
Institutional collaboration and publication analysis. **(A)** Publication output of institutions with more than 100 publications, ranked according to the total number of published studies. The figure highlights the leading institutions contributing to the pEVs research field. **(B)** Organizational Collaboration Diagram. Each node represents an institution, with node size proportional to publication output, while the connecting lines indicate collaborative relationships between institutions. Different colors represent distinct collaboration clusters, reflecting closely connected research groups. **(C)** Institutional Collaboration Density Map. Areas with warmer colors (yellow/red) indicate institutions with higher publication density and stronger collaborative activity, whereas cooler colors (green/blue) represent lower research activity. The density map demonstrates the major institutional hubs and collaborative hotspots in global pEVs research.

Bibliometric analysis at the institutional level enables the identification of highly productive and influential research organizations while illustrating patterns of inter-institutional collaboration. These findings may help researchers identify potential partners for cross-regional cooperation, promote the integration of complementary resources and expertise, and foster higher-quality research outcomes. A total of 4, 791 institutions participated in the study. **Figure 4A** shows the 19 institutions that published more than 100 papers. The top two institutions published over 200 papers each: Institut National de la Santé et de la Recherche Médicale (n = 246) and Harvard University (n = 202). **Figure 4B** and **Figure 4C** display the 171 institutions with more than 12 publications (excluding one isolated node with no collaborations), which form five collaborative clusters. Cluster 1 (green) constitutes the global core collaboration network, with Harvard Med Sch as the largest node and the one with the widest connectivity, serving as the key institution in this cluster. **Figure 4C** shows that there are multiple high-density hotspots in the network, centered around institutions such as Harvard Med Sch, Shanghai Jiao Tong Univ, and Univ Milan, exhibiting high concentration. Institutions that participated in the research earlier are mainly concentrated in Europe, while research in China and the Asia-Pacific region began relatively later. The distribution of the five collaborative clusters indicates that a relatively stable collaborative framework has emerged among research institutions, both geographically and academically; however, the intensity of collaboration varies across the clusters.

### 3.4 Core journals and publication patterns

**Figure 5:**
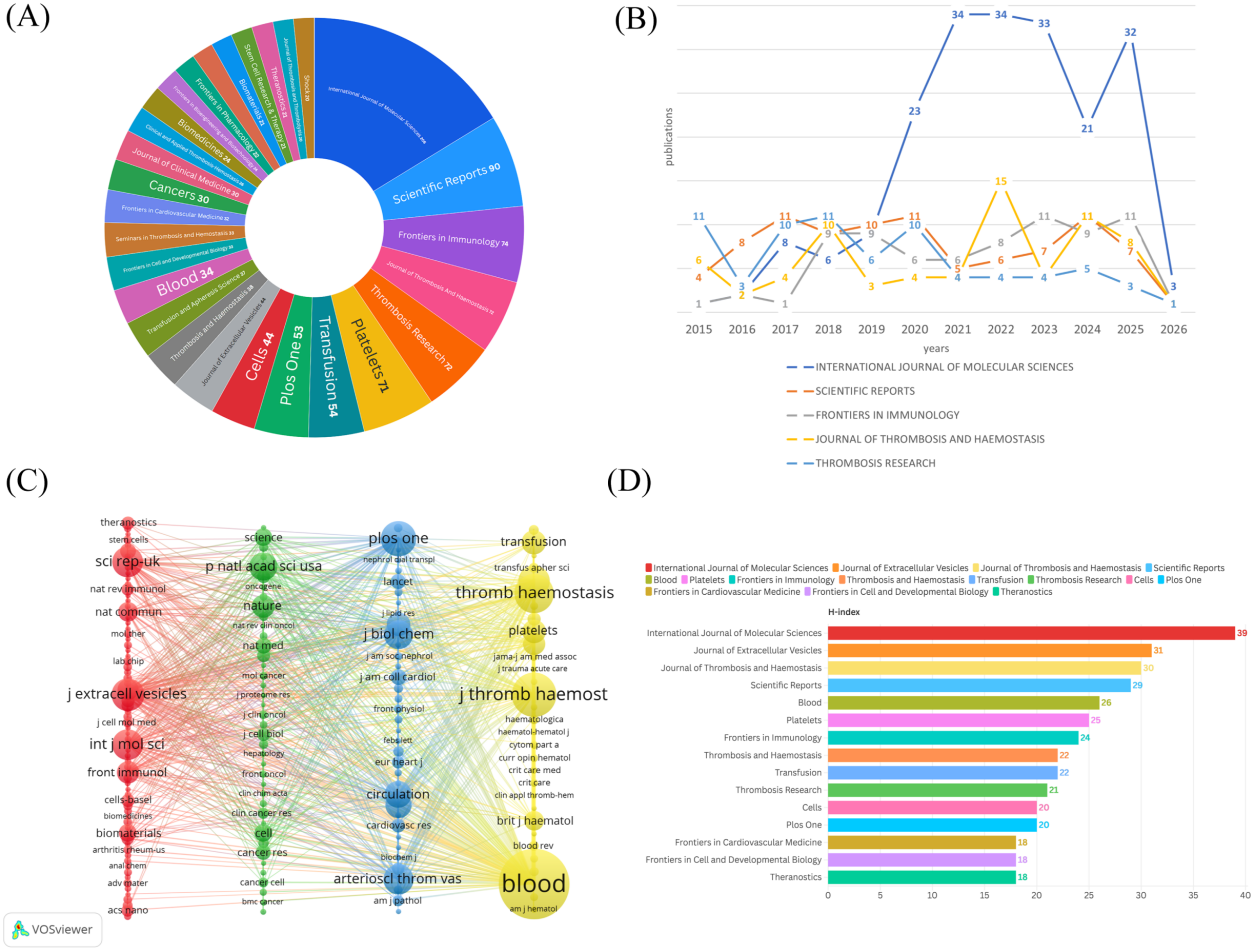
Journal publication distribution, trends, and citation impact analysis. **(A)** Pie chart showing the publication output of journals with more than 20 published articles. The area of each circle is proportional to the corresponding publication output. Font sizes were adjusted solely to improve label readability and accommodate differences in journal name length, and therefore do not represent publication quantity or any other quantitative metric. **(B)** Annual publication trends of the top five journals ranked by publication volume from 2015 to 2026. The figure demonstrates the temporal changes in publication output and the evolving contribution of leading journals to pEVs research over time. **(C)** Citation Network of Journals. Each node represents a journal, with node size proportional to citation frequency, while the connecting lines indicate citation relationships among journals. Different colors represent distinct journal clusters, reflecting closely related research topics and citation patterns.**(D)** Ranking of the top 15 journals according to H-index. The bar chart presents the academic impact and influence of major journals in the pEVs research field, with higher H-index values indicating greater scientific influence and citation performance.

Bibliometric analysis of journals not only helps identify the major publication venues and the distribution of academic influence within a research field, but also reveals the interdisciplinary nature of emerging research topics. A total of 1, 163 journals were included in the study. The results show that the literature is primarily concentrated in a small number of journals. As shown in **Figure 5A** and **Figure 5B**, *International Journal of Molecular Sciences* had the highest number of publications (206), followed by *Scientific Reports* (90 articles). The number of articles published in *International Journal of Molecular Sciences* has grown rapidly since 2019, peaking between 2021 and 2023, making it the primary contributor to the recent expansion of the pEV literature. In contrast, the number of articles in *Scientific Reports* and *Frontiers in Immunology* has remained relatively stable or shown a slow increase.

**Figure 5C** shows that the 172 journals with more than 12 citations form four clusters. These journals primarily cover the following fields: mechanisms of thrombosis and hemostasis, hematological diseases, basic biological research and oncology, and Evs and basic molecular biomedicine, while also encompassing interdisciplinary areas such as immunology and biomaterials, as well as stem cell diagnosis and therapy. The largest and most tightly connected cluster is the yellow one. With a large number of nodes, dense connections, and high centrality, this cluster exerts significant influence on research trends and academic development in the field. **Figure 5D** displays the 15 journals with an H-index greater than 18. *International Journal of Molecular Sciences* maintains the highest output while also possessing a high H-index, whereas *Journal of Extracellular Vesicles* demonstrates considerable influence despite a relatively limited number of publications.

### 3.5 Leading authors and author collaboration networks

**Figure 6:**
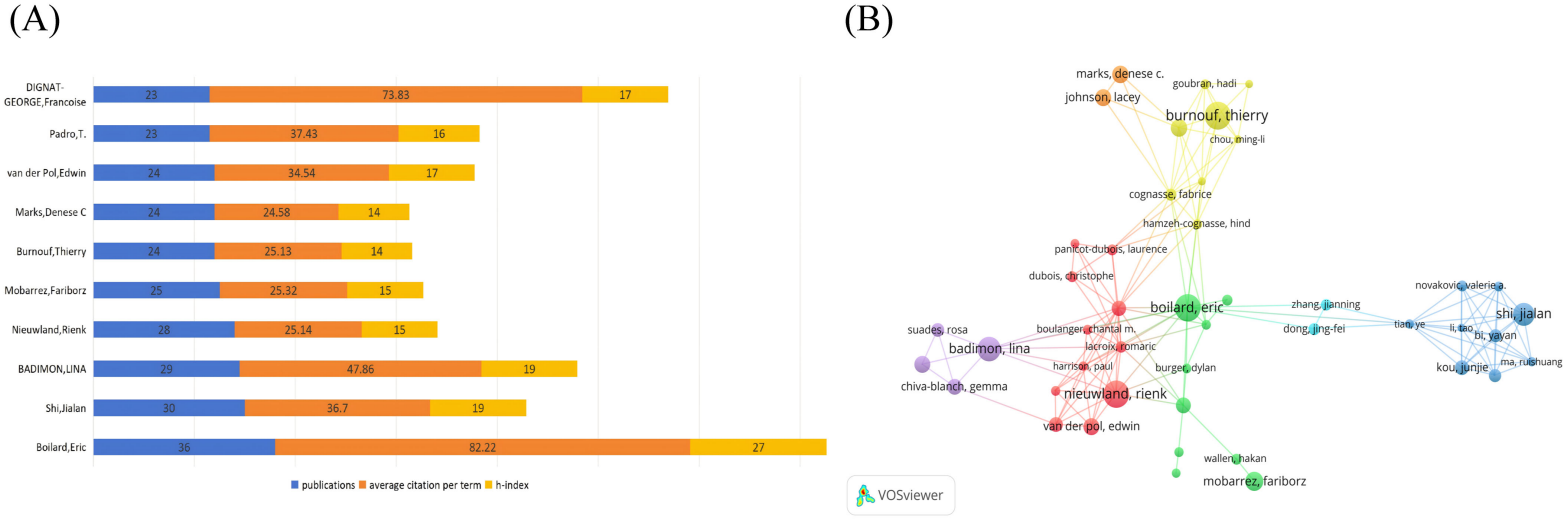
Author productivity and collaboration network analysis. **(A)** Productivity and academic impact analysis of the top 10 most prolific authors in the field. The stacked bar chart presents the total number of publications for each author, together with their corresponding H-index and average citations per article, thereby reflecting both research productivity and scientific influence. **(B)** Author Collaboration Network. Each node represents an individual author, with node size proportional to publication output, while the connecting lines indicate co-authorship relationships between authors. Different colors represent distinct collaborative clusters, highlighting major research teams and cooperative relationships within the global pEVs research community.

Author analysis helps identify the key contributors within a research field and reveal patterns of scientific collaboration. It provides valuable insights into academic influence and productivity while helping researchers identify potential mentors, collaborators, and leading research groups, thereby facilitating knowledge exchange and promoting the development of the field. **Figure 6A** shows that Eric Boilard from Laval University in Canada ranks first with 36 publications, while Shi, Jialan, and Lina Badimon are tied for second place with 26 publications each, indicating that they are among the leading contributors in this field. Eric Boilard not only leads in terms of publication volume, but also tops the list in terms of H-index (22) and average citations per paper (82.22, significantly higher than the overall average for the field included in this study). This indicates that while Eric Boilard’s team maintains a high output, the quality of their research remains strong and has garnered widespread recognition from peers both domestically and internationally.

The 77 authors who published at least 10 articles formed seven distinct collaboration clusters (**Figure 6B**). The clusters are clearly defined and exhibit a certain degree of dispersion. Although these clusters showed relatively clear boundaries, numerous inter-cluster connections were observed, suggesting the existence of stable collaborative relationships among major research groups in the field. Among these, the collaborative cluster centered on Eric Boilard serves as a key node in establishing cooperative relationships with other clusters across the entire network. The cluster centered on Shi Jialan exhibits distinct geographical characteristics. The team consists primarily of Chinese scholars and maintains close and stable internal collaboration. However, it exhibits strong links only with the cluster centered on Eric Boilard and has relatively weak connections with other clusters in Europe and the United States, indicating a lack of cross-regional collaboration.

### 3.6 Thematic evolution and research trends revealed by LDA topic modeling

**Figure 7:**
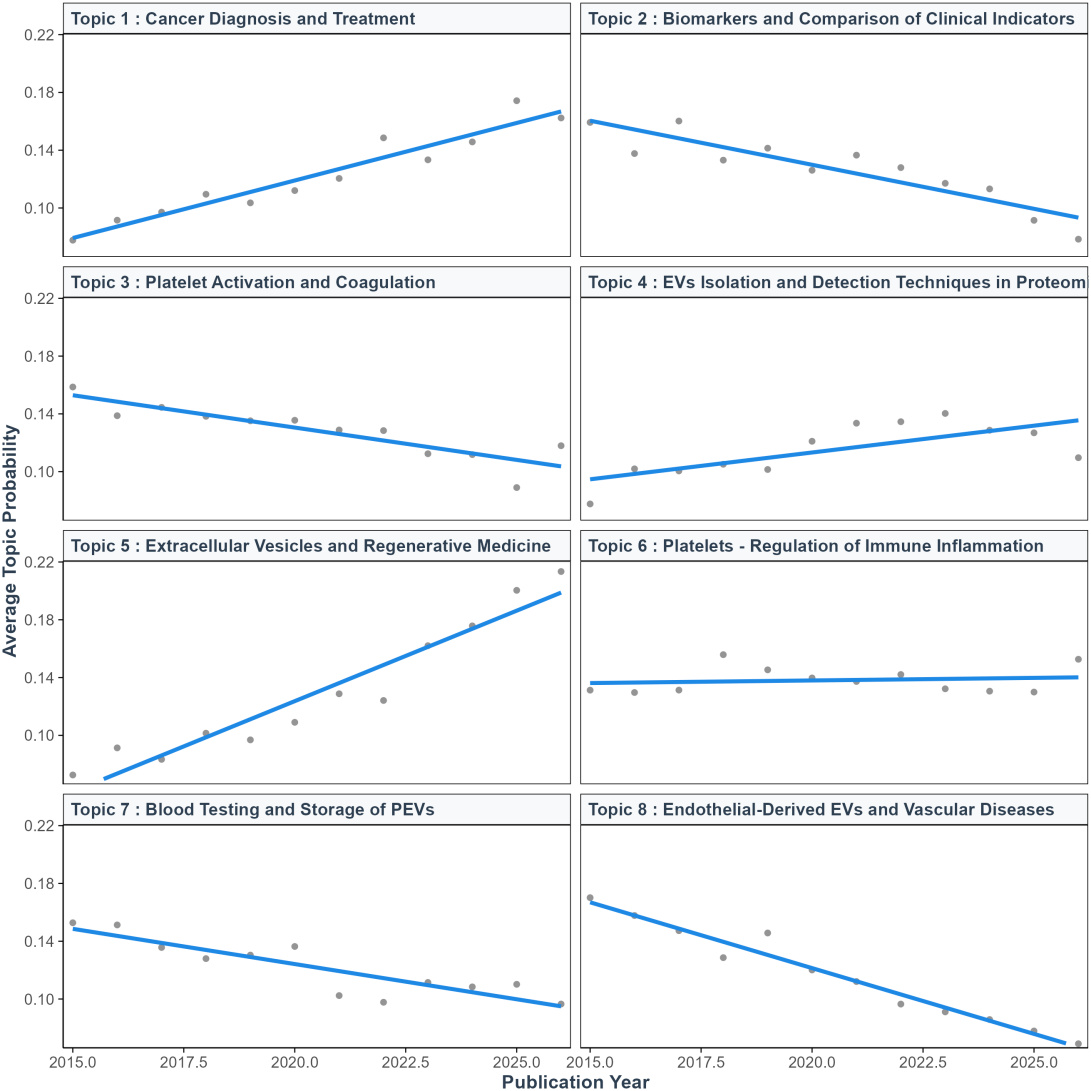
Trends in Potential Themes in Research on Platelet-Derived Extracellular Vesicles (2015–2026). The figure shows the annual average posterior probability for eight themes, along with annual data points and the fitted linear trend.

LDA topic modeling was employed to identify underlying thematic structures within the pEV literature and to characterize the temporal evolution of research focuses. Compared with traditional keyword-based approaches, LDA captures broader conceptual relationships across publications, providing a more comprehensive overview of emerging research directions. The LDA model identified eight major latent themes from 2, 897 articles. The prevalence of each theme ranged from 0.11 to 0.14, with a relatively balanced distribution. No single theme was found to be overwhelmingly dominant (**Figure 7**).

Based on thematic characteristics, the eight themes could be grouped into three major categories. The “Basic Mechanisms Research” category included Topic 3 (Platelet Activation and Coagulation, Prevalence=0.13), Topic 6 (Platelets - Regulation of Immune Inflammation, Prevalence=0.14), and Topic 8 (Endothelial-Derived EVs and Vascular Diseases, Prevalence=0.11), which focus respectively on pEV-mediated regulation of coagulation and thrombosis, modulation of immune inflammation, and the pathophysiological mechanisms of vascular diseases. These represent early research mechanisms in this field. The “Technical Methods” category included Topic 4 (EVs Isolation and Detection Techniques in Proteomics, Prevalence=0.12) and Topic 7 (Blood Testing and Storage of PEVs, Prevalence=0.12), addressing key technical issues such as the isolation and purification of pEVs, optimization of detection techniques, and sample storage. “The Clinical Translation and Application” category included Topic 1 (Cancer Diagnosis and Treatment, Prevalence=0.13), Topic 2 (Biomarkers and Comparison of Clinical Indicators, Prevalence=0.13), and Topic 5 (Extracellular Vesicles and Regenerative Medicine, Prevalence=0.13), highlighting the translational application of pEVs in cancer, biomarker research, and regenerative medicine. (**Table 1**) The results of the thematic time-series analysis of the linear regression model show that the eight themes can be classified into three trend categories: themes exhibiting an upward trend include Themes 1, 4, 5, all with P-values < 0.05, representing recent research hotspots in the field of pEVs. Themes exhibiting a downward trend include Themes 2, 3, 7, and 8, all with P-values < 0.05 and coefficients of variation (CV) > 10%, indicating a sustained decline in overall research interest. Theme 6 shows no significant temporal trend (P = 0.661) and has remained stable over the long term.

**Table 1:** Topics discovered from 2897 articles published between 2015 and 2026.

Topic	Prevalence	Top terms	Label
Topic1	0.13	cancer, tumor, cells, circulating, treatment, liquid, potential, biopsy	Cancer Diagnosis and Treatment
Topic2	0.13	patients, levels, higher, compared, significantly, disease, controls, healthy	Biomarkers and Comparison of Clinical Indicators
Topic3	0.13	platelet, activation, platelets, thrombosis, coagulation, activity, platelet_activation, factor	Platelet Activation and Coagulation
Topic4	0.12	evs, extracellular, vesicles, extracellular_vesicles, plasma, ev, proteins, vesicles_evs	EVs Isolation and Detection Techniques in Proteomics
Topic5	0.13	exosomes, cells, cell, growth, expression, stem, healing, wound	Extracellular Vesicles and Regenerative Medicine
Topic6	0.14	cells, platelets, immune, cell, inflammation, diseases, inflammatory, release	Platelets - Regulation of Immune Inflammation
Topic7	0.12	blood, platelet, microparticles, platelets, plasma, storage, methods, flow	Blood Testing and Storage of PEVs
Topic8	0.11	endothelial, microparticles, mps, vascular, pmps, increased, endothelial_cells, cells	Endothelial-Derived EVs and Vascular Diseases

**Figure 8:**
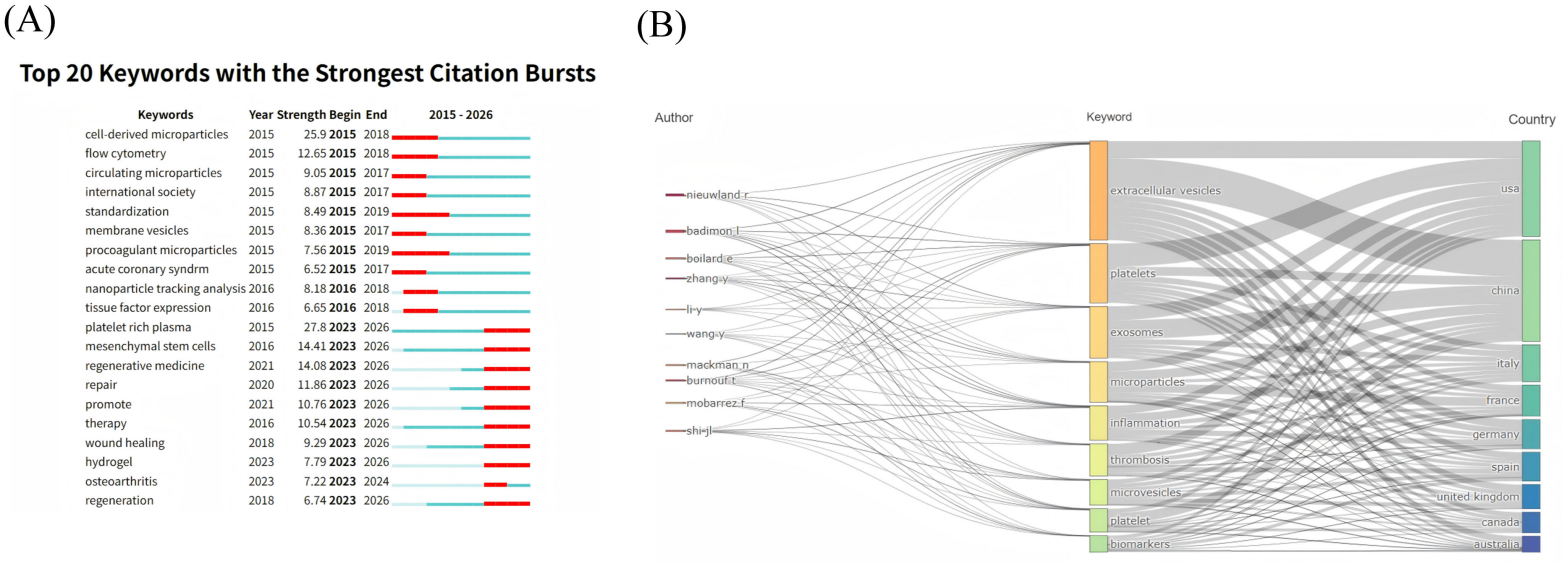
Keyword distribution and author-keyword-country relationship analysis. **(A)** Visualization of the top 20 keywords. The table provides each keyword’s burst strength, start year, and end year of the burst. Red bars mark the time period of citation burst, blue bars represent the full study period. **(B)** “Author-Keyword-Country” Sankey diagram. This diagram visualizes the association patterns between core authors, high-frequency keywords, and major contributing countries. The width of the connecting bands reflects the strength of the association between paired nodes.

To evaluate the robustness of LDA-based topic identification, the results were cross-validated using CiteSpace keyword emergence analysis. The results showed that the two analytical methods were highly consistent regarding the direction of topic evolution (**Figure 8A**). Early-emerging keywords (2015–2017), such as “cell-derived microparticles, “ “flow cytometry, “ and “standardization, “ all corresponded to the LDA analysis results for the underlying mechanisms (Theme 3) and methodology (Themes 4 and 7) identified by the LDA analysis, representing the primary research directions in the early stages of the pEVs field. In contrast, keywords emerging between 2021 and 2023, such as “regenerative medicine, “ “therapy, “ and “healing, “ aligned closely with the increasing Topic 5, indicating a gradual shift toward clinical translation and regenerative medicine applications.

An “author–keyword–country” Sankey diagram was constructed to further characterize the research landscape and cross-validate the LDA findings (**Figure 8B**).

Researchers such as Nieuwland R, Badimon L, and Boilard E were primarily associated with keywords including “platelets, “ “thrombosis, “ and “microparticles, “ corresponding mainly to Topics 3 and 8. Scholars such as Zhang Y, Li Y, Wang Y, and Shi JL exhibit wider connection bands with nodes such as “exosomes, “ “inflammation, “ and “biomarkers, “ corresponding to multiple research themes in the LDA model, including Themes 1, 2, 5, and 6. This reflects the diverse research directions of these scholars in the field of pEVs. China and the United States exhibited the broadest keyword connections, indicating extensive participation across multiple research directions in the pEV field.

### 3.7 Current landscape of clinical translation

The 30 included clinical studies primarily focused on five aspects of disease: pathophysiological functions, biomarker applications, clinical interventions and regulation, therapeutic translation, and the source and administration strategies of EVs.

#### 3.7.1 The pathophysiological functions of pEVs

Accumulating evidence indicates that pEVs contribute to coagulation, inflammation, and tissue repair. Studies have shown that the procoagulant activity of EVs is closely associated with surface-exposed phosphatidylserine (PS), which promotes thrombin generation and fibrin clot formation (13, 14). In patients with transfusion-dependent β-thalassemia, there is a significant increase in PS-positive pEVs, which is associated with the development of a hypercoagulable state and, consequently, the onset of pulmonary arterial hypertension (PAH) (15). Following bone marrow transplantation, improvements in hypercoagulability are accompanied by reductions in activated platelets and pEV levels (16). In addition, the 75-nm nanofiltration process, which removed nanosized EV populations including pEVs, significantly reduced the *in vitro* procoagulant activity of plasma (14).

Circulating pEVs are also involved in inflammatory regulation. In systemic lupus erythematosus, immunoglobulin G (IgG)-positive pEVs can activate monocytes and promote the release of pro-inflammatory cytokines, including interleukin-1β (IL-1β) and tumor necrosis factor-α (TNF-α) (17). In patients with stable cardiovascular disease treated with rivaroxaban combined with aspirin, pEVs levels decreased significantly, and the expression profile of pro-inflammatory proteins associated with circulating EVs was also downregulated (18). In addition, elevated circulating pEV levels in multiple sclerosis have likewise been associated with chronic platelet activation, systemic inflammation, and thrombotic risk (19).

In regenerative medicine, activated pEVs contain multiple bioactive factors, including insulin-like growth factor (IGF) and transforming growth factor-β (TGF-β). Clinical studies have shown that these pEVs can induce the proliferation and migration of dermal fibroblasts while enhancing the angiogenic capacity of dermal endothelial cells (20). Platelet-derived preparations containing pEVs were associated with improved facial skin appearance through the promotion of dermal collagen and elastin synthesis (21).

#### 3.7.2 The application of pEVs as biomarkers

Clinical studies suggest that pEVs may serve as potential biomarkers in hematological and cardiovascular diseases. In β-thalassemia, circulating pEV levels reflect hypercoagulability and are associated with PAH development, while dynamic changes in pEV levels after bone marrow transplantation correlate with recovery of coagulation function (15, 16).

In the field of cardiovascular disease, elevated levels of tissue factor-positive pEVs (historically described as CD142+/CD61+ microparticles) in high-risk populations were associated with an increased risk of cardiovascular events during a four-year follow-up period. A predictive model incorporating platelet-, endothelial-, and leukocyte-derived EVs achieved an area under the receiver operating characteristic curve (AUC) of 0.805, demonstrating good predictive performance (22).

#### 3.7.3 The effect of clinical interventions on pEVs

Multiple randomized controlled trials have demonstrated that clinical interventions can influence the levels and function of pEVs, with varying effects observed among different interventions. Regarding pharmacological treatment, the addition of dietary nitrates to clopidogrel therapy in patients with coronary heart disease can further reduce pEV levels (23). With regard to non-pharmacological interventions, levels of pEVs increased in patients with obstructive sleep apnea after discontinuing continuous positive airway pressure (CPAP) therapy for 2 weeks (24). However, 12 months of regular exercise training did not significantly increase pEVs levels in patients with diabetes and coronary heart disease (25).

#### 3.7.4 Clinical studies on the therapeutic applications of pEVs

The world’s first Phase I clinical trial (double-blind, placebo-controlled) of allogeneic pEVs in humans, published in 2023, demonstrated that clinical-grade pEVs purified using the LEAP process were safe and well-tolerated when administered via injection in a wound healing model in healthy subjects, laying the groundwork for subsequent efficacy studies targeting chronic, hard-to-heal wounds (20).

In clinical settings, human platelet extracts containing pEVs have undergone initial clinical translation in the field of aesthetic medicine. After 12 weeks of continuous topical application, the thickness of dermal collagen fibers increased significantly, and issues such as facial skin aging and hyperpigmentation were improved (21). In the field of transfusion medicine, the levels of pEVs in machine-collected platelet products are associated with functional impairment during platelet storage (26).

#### 3.7.5 Sources and administration strategies of pEVs

The clinical translation of pEVs is influenced not only by their biological properties but also by factors such as source, route of administration, and treatment regimen. Clinical studies have explored both autologous and allogeneic pEVs. At present, most studies in regenerative medicine and aesthetic medicine use pEVs derived from patients’own platelet-rich plasma (PRP) or platelet extracts because of their convenient availability, favorable immunocompatibility, and compatibility with existing PRP-based therapeutic approaches (27). However, autologous products remain susceptible to inter-individual variability in preparation procedures, source material quality, and batch consistency, which may limit their large-scale clinical application. In contrast, allogeneic pEVs offer greater potential for standardized manufacturing. Given the broad availability of platelet sources, including blood bank-derived platelet concentrates and near-expiry platelet products, allogeneic pEVs could be produced under Good Manufacturing Practice (GMP) conditions with centralized manufacturing, quality control, and batch standardization (28).

Notably, the clinical translation of allogeneic pEVs has already shown encouraging progress. The first-in-human phase I clinical trial conducted by Johnson et al. demonstrated that locally administered allogeneic pEVs were safe and well tolerated in the treatment of delayed wound healing (23), providing a foundation for future efficacy studies. These findings suggest that although autologous pEVs remain the predominant approach in current clinical research, allogeneic pEVs may represent an important direction for the future development of standardized and scalable EV-based therapeutics.

At present, local administration remains the most commonly used delivery strategy, including intradermal injection, intralesional injection, and topical application. These approaches are primarily employed for wound healing, cartilage repair, and skin rejuvenation. In addition, several studies have reported the therapeutic potential of hydrogel-based formulations, such as pEV-loaded hydrogels or creams, for cutaneous wound repair (28). By comparison, studies involving intravenous administration remain relatively limited, although this route has been explored in investigations of systemic diseases and biodistribution (29, 30).

Considerable heterogeneity also exists in treatment regimens across studies. Different studies have adopted diverse approaches to define pEV dosage, including particle concentration or protein content. However, detailed dosing information is frequently unavailable or incompletely reported in published studies. The reported dosing strategies therefore vary substantially across studies, highlighting the current lack of standardized dosing criteria for clinical applications (27, 28). Substantial variation is also observed in dosing frequency, including single local injections, weekly repeated administrations, multi-cycle treatment schedules, and protocols combining injections with topical application (13, 31). Current evidence remains insufficient to determine the optimal dose, administration frequency, or treatment regimen for pEV-based therapies, highlighting that the clinical translation of pEVs is still at an early stage. Furthermore, no standardized dosing framework for pEV administration has yet been established, making direct comparisons of therapeutic efficacy across studies challenging.

## 4 Discussion

### 4.1 Overall research landscape and development trends

This study combines traditional bibliometrics with LDA topic modeling to systematically analyze the research landscape of pEVs from 2015 to 2026, revealing a rapid increase in the number of publications from 2015 to 2026. In 2021 in particular, the number of publications reached its first peak (n=448), and the number of reviews increased accordingly. On the one hand, this may be attributed to the rapid development of the life sciences driven by the COVID-19 pandemic, as researchers needed to quickly synthesize a large volume of research findings (32, 33); on the other hand, in the three years following the release of the MISEV 2018 guidelines, researchers conducted their work in accordance with established standards. The concentrated emergence of a large number of well-structured research findings subsequently drove the need to summarize progress in the field.

China and the United States are the two countries with the highest publication output and the closest collaboration, and they have made significant contributions to this field. However, despite their comparable publication outputs, notable differences were observed in terms of collaborative networks and scientific influence. The United States exhibited substantially higher network centrality than China and occupied a more prominent position within the global collaboration network. China maintained close collaborative relationships primarily with a few countries, such as the United States and Australia, while cooperation with European nations remains insufficient, which to some extent limits the international dissemination of research findings. This pattern was further supported by the author collaboration network, which showed that the Chinese research cluster centered on Shi Jialan remained relatively fragmented and lacked extensive cross-regional collaboration. Although China has experienced rapid growth in publication output, likely driven by substantial national investment in biomedical research over recent years, its average citation impact remains lower than that of the United States. In addition to the relatively later development of this field in China, a more concentrated collaboration structure may have limited access to broader international research networks, thereby reducing opportunities for knowledge exchange and citation visibility. Institutions such as Harvard Medical School and the University of Pennsylvania occupy central positions in the global collaboration network. In China, high-output institutions such as Shanghai Jiao Tong University and Harbin Medical University are currently focused primarily on participating in international collaborations and have not yet developed the industry-leading capabilities seen in U.S. institutions.

*International Journal of Molecular Sciences* has seen a rapid increase in the number of publications since 2019, becoming a major source of research output in this field. Although *Journal of Extracellular Vesicles* publishes a limited number of articles, it wields significant influence in the field of extracellular vesicle research due to its high h-index. The co-citation network of journals further indicates that pEV research is highly interdisciplinary. It integrates knowledge from thrombosis and hemostasis, hematology, oncology, immunology, biomaterials, and regenerative medicine. This finding suggests that the field has moved beyond traditional platelet biology and is emerging as a frontier at the intersection of EV basic science and translational medicine. Eric Boilard has made significant contributions, with his team’s research covering key areas such as the role of platelets in the immune system and pEVs in chronic inflammation (34, 35). Through collaboration with other research groups, he has built bridges of knowledge that have influenced the trajectory of development in the field of pEVs.

### 4.2 Current status and challenges of clinical translation

Regenerative medicine and oncology have emerged as the two most active research areas in the pEV field since 2021. However, the number of related clinical trials remains limited, highlighting a substantial gap between basic research and clinical translation. This discrepancy likely arises from multiple factors. For example, the tumor models and acute skin injury models in immunodeficient mice commonly used in preclinical studies differ fundamentally from the immunosuppressive tumor microenvironment and the complex pathology of chronic non-healing wounds in humans. As a result, promising findings observed in animal models may not be readily reproduced in clinical settings. In addition, the clinical translation of pEVs faces several practical challenges, including large-scale manufacturing, long-term storage stability, sterility control, and batch-to-batch consistency. These issues continue to hinder the reliable production and broader application of clinical-grade pEV products.

Current clinical evidence indicates that the procoagulant properties of pEVs have been demonstrated across a range of disease settings, and changes in pEV levels may reflect the therapeutic effects of certain antithrombotic treatments, such as rivaroxaban combined with aspirin (23). However, several limitations remain. In the vast majority of clinical studies, pEV levels have been assessed only as non-primary observational indicators, and no clinical trial has yet adopted strategies specifically targeting the generation or function of pEVs. Future studies are needed to systematically compare the procoagulant properties of pEVs across different diseases and intervention conditions in order to identify key pathogenic targets and provide clearer directions for clinical intervention strategies. In addition, studies investigating pEVs as therapeutic agents remain limited to phase I safety trials. Evidence regarding their clinical efficacy, optimal dosage range, administration frequency, and long-term safety is still scarce. Furthermore, clinical trials have not yet comprehensively compared differences in the procoagulant potential of pEVs across various disease settings or therapeutic interventions. Notably, the preliminary application of human platelet extracts containing pEVs in aesthetic medicine (21)highlights their translational potential and suggests possible future clinical and commercial value. Nevertheless, whether the regenerative effects observed in preclinical studies can be consistently reproduced in humans under complex pathological conditions remains uncertain and requires validation through additional high-quality clinical trials.

Beyond the limited clinical evidence, regulatory requirements represent another major barrier to the clinical translation of pEVs. Although EV research has expanded rapidly in recent years, a unified regulatory framework for EV-based products has not yet been fully established. At present, most regulatory agencies classify EVs as biological products or advanced therapy medicinal products (ATMPs). Consequently, their clinical development is generally expected to comply with existing regulations governing cell therapies, biological products, and sterile medicinal products (36, 37). However, the heterogeneity of EVs in terms of source, composition, and biological function continues to complicate the establishment of standardized approaches for potency testing, dose definition, and long-term safety evaluation (37, 38).

Currently, the MISEV2023 guidelines published by ISEV remain the most widely recognized methodological reference for EV research. These guidelines provide comprehensive recommendations on EV nomenclature, isolation, characterization, and reporting standards. In addition, recent studies focusing on the quality and safety of EV-based therapeutics have further emphasized the importance of manufacturing, storage, transportation, and quality control requirements for clinical-grade EV products (38).

From a regulatory perspective, the U.S. Food and Drug Administration (FDA) generally regulates EV-related products within the framework of biological products. Therefore, investigators planning clinical studies may refer to FDA guidance documents related to biologics, cell-based therapies, and Chemistry, Manufacturing, and Controls (CMC) requirements (39). Similarly, the existing European Medicines Agency (EMA) framework for ATMPs provides important guidance for the GMP production, sterility assurance, and traceability of EV-based therapeutics (36, 37). Consequently, establishing manufacturing and quality control systems that meet regulatory expectations at an early stage of clinical development is essential. Both ISEV and international regulatory agencies have emphasized several key requirements for the clinical translation of EV products, including source traceability, process standardization, batch-to-batch consistency, sterility control, endotoxin testing, and the assessment of EV purity and potency (6, 36). Accordingly, EV manufacturing processes that comply with GMP standards are increasingly regarded as a prerequisite for clinical investigation.

Despite these advances, the clinical translation of EV-based products continues to face several important challenges, including insufficient manufacturing standardization, difficulties in controlling batch-to-batch variability, the lack of universally accepted potency assays, and limited data on long-term storage stability (36, 38). Therefore, future clinical studies of pEVs should not only adhere to technical guidelines such as MISEV2023 but also incorporate regulatory principles established by agencies such as the FDA and EMA. The gradual development of standardized regulatory and quality-control frameworks tailored to EV products will be essential for achieving safe, reproducible, and scalable clinical applications.

### 4.3 Mechanistic insights and translational progress in pEV research

From a methodological perspective, LDA topic modeling and CiteSpace burst word analysis capture different dimensions of research dynamics. LDA tracks changes in the overall probability distribution of latent topics to reflect long-term thematic evolution. In contrast, CiteSpace uses Kleinberg’s burst detection algorithm, which analyzes individual keywords to identify sudden spikes in frequency over short periods. Core terms of Topic 1, including cancer and tumor, are among the most frequently used terms in biomedical research. Their increasing prominence in the pEVs field reflects a gradual rise in usage within the research context, rather than a short-term burst. This pattern does not meet the criteria for burst word identification and differs fundamentally from the development patterns of the top 20 emerging keywords identified in this study.

Regenerative medicine and clinical translation represent the most prominent current hotspots in pEV research. These themes show continuously increasing topic strength in the LDA analysis and are supported by emerging keywords such as tissue repair, extracellular vesicles, and translational applications. In contrast, cancer diagnosis and therapy show a significant and sustained growth in annual topic probability, indicating a persistent and increasing research interest. Although cancer-related terms do not appear among the high-intensity burst keywords, this likely reflects that”cancer” and”tumor”are already high-frequency terms in biomedical research, so their prominence increases gradually rather than abruptly. Conversely, several mature topics, including thrombosis, inflammation, biomarker studies, and vascular diseases, show a relative decline in prominence but remain important components of the field.

#### 4.3.1 pEVs in oncology: mechanistic advances

Theme 1 of the LDA analysis indicates a rising trend in the study of pEVs in oncology research. As important mediators of the interaction between platelets and the tumor microenvironment, pEVs play a key role in regulating tumor proliferation, metastasis, metabolism, and immune evasion. In recent years, research on pEVs has also made progress in the areas of tumor diagnostic biomarkers and engineered drug delivery systems (40).

The p38 mitogen-activated protein kinase (MAPK) signaling pathway is one of the mechanisms by which pEVs regulate tumor cell migration and invasion; this pathway has been observed to play a role in both breast cancer and colorectal cancer (41, 42). In breast cancer, pEVs promote cell migration by modulating intracellular calcium signaling and activating p38 MAPK and myosin light chain kinase (42). In colorectal cancer, pEVs upregulate the expression and activity of matrix metalloproteinase-2 (MMP-2) and matrix metalloproteinase-9 (MMP-9) through this pathway, thereby enhancing cellular invasiveness (41). In addition, pEVs can promote the malignant transformation of breast and colorectal cancer cells by carrying integrin molecules or inducing the expression of genes associated with epithelial-mesenchymal transition (43, 44). It is worth noting that pEVs released by platelets activated via different receptors exhibit varying abilities to promote melanoma proliferation, invasion, and angiogenesis (45). This suggests that the activation state of platelets may be a key factor influencing the functional heterogeneity of pEVs.

With regard to the remodeling of the tumor microenvironment, a subset of pEVs containing functional mitochondria can transfer these mitochondria to breast cancer cells, increasing adenosine triphosphate (ATP) production in the recipient cells and thereby enhancing their migratory and invasive capabilities (46). These mitochondrial transfers can induce metabolic reprogramming in tumor cells, promoting their proliferation and enhancing their resistance to chemotherapy drugs (47, 48). In addition, pEVs can influence tumor progression by delivering molecules such as leucine-rich α-2 glycoprotein 1 (LRG1) and LINC00183, or by regulating the expression of cell cycle-related proteins (44, 49, 50). The regulation of tumor cells by pEVs involves at least the mechanisms described above; however, the relative importance and synergistic effects of these mechanisms in promoting tumor progression remain unclear, and the specific contributions of each mechanism to phenotypes such as proliferation, migration, and chemoresistance have yet to be quantified. Clarifying these points will aid subsequent research in identifying the dominant mechanisms or common points of convergence underlying pEV-mediated tumor function, thereby providing a basis for selecting targets for intervention strategies.

PEVs also exhibit complex effects in tumor immune regulation. They can transfer integrin β3 to tumor-infiltrating T cells, thereby enhancing the T cells’ antitumor immune response (51). However, pEVs may also promote the formation of an immunosuppressive microenvironment by influencing myeloid suppressor cells or modulating macrophage polarization (52). With regard to angiogenesis, activated pEVs can stimulate lung cancer cells to express pro-angiogenic factors such as vascular endothelial growth factor, hepatocyte growth factor, and interleukin-8 (53, 54). These findings suggest that the specific effects of pEVs in tumor immunity depend on their source, activation status, and the type of recipient cells.

In recent years, pEVs have also attracted increasing attention as potential biomarkers in tumor liquid biopsy. Multiple studies have shown that changes in circulating pEV levels and their contents are closely associated with tumor progression and prognosis. In patients with non-small cell lung cancer, elevated levels of circulating pEVs are significantly associated with disease progression and resistance to immunotherapy (55, 56). Elevated expression levels of LINC00183 (44) in pEVs from colorectal cancer patients and LRG1 (50) in multiple myeloma patients are both closely associated with poor patient prognosis. In patients with hormone-resistant prostate cancer, pEVs concentration is associated with overall survival (57). Patients with type 2 diabetes and breast cancer exhibit altered miRNA profiles in their pEVs, which can promote the invasion of triple-negative breast cancer cells (58). These findings support the potential utility of pEVs as minimally invasive biomarkers for tumor monitoring and prognostic assessment.

Due to their inherent targeting ability, low immunogenicity (59), and potential for engineering, pEVs are being explored as a potential tumor drug delivery system. PEVs can load doxorubicin, thereby enhancing their cytotoxic effect on tumor cells (60). PEVs prepared from expired platelet concentrates and loaded with paclitaxel effectively inhibit the migration, invasion, and angiogenesis of breast cancer cells (61). In addition, a biomimetic nanoplatform based on platelet membranes has demonstrated excellent tumor-targeting capabilities; the nanoparticles can also be loaded with active ingredients from traditional Chinese medicine, such as curcumin, to enable targeted therapy against melanoma cells (62, 63). In addition, engineered platelet-based therapeutic platforms have demonstrated potential for enhancing antitumor immunity and overcoming treatment resistance. For example, pEV-mediated targeting of myeloid cells following anti-programmed death-ligand 1 (PD-L1) therapy was shown to reverse acquired immune resistance and improve the efficacy of subsequent immunotherapy (52). Similarly, a platelet-based degradation platform delivering heat shock protein 90 (HSP90)-ligand complexes efficiently degraded bromodomain-containing protein 4 (BRD4) within tumor cells or PD-L1 on the cell surface, thereby suppressing postoperative tumor recurrence and metastasis (64).

#### 4.3.2 pEVs in regenerative medicine: mechanistic advances

Traditional regenerative therapies (such as stem cell therapy and the administration of recombinant growth factors) have significant limitations in terms of donor sourcing, immune rejection, ethical concerns, short *in vivo* half-lives, and clinical accessibility; as a result, cell-free therapies have emerged as a key direction in regenerative medicine. Due to their unique biological properties, pEVs have become a hot topic of research in the field of regenerative medicine.

PEVs can precisely target damaged sites using their surface markers. As multifunctional carriers, they can load and deliver various bioactive substances, such as vascular endothelial growth factor (VEGF), platelet-derived growth factor (PDGF), and miR-126, to synergistically promote angiogenesis and cell proliferation while regulating inflammation, thereby exerting therapeutic effects (65). PEVs can also be combined with smart materials. For example, when co-encapsulated with reduced graphene oxide (rGO) in a hydrogel, they not only enable on-demand release triggered by near-infrared light but also synergistically enhance the pEVs’ ability to promote re-epithelialization, regulate macrophage polarization, and scavenge reactive oxygen species in diabetic wounds, thereby effectively promoting tissue regeneration (66). In the field of cartilage repair, EVs isolated from platelet-rich plasma (PRP-EVs) have been reported to recruit endogenous stem cells, promote chondrogenic differentiation, and modulate inflammatory pathways, thereby representing a promising cell-free therapeutic strategy for osteoarthritis (36). It is worth noting that, although studies have revealed the detrimental role of pEVs in driving pathological processes in sepsis, this indirectly underscores their potent regulatory capacity as intercellular messengers, thereby providing a theoretical basis for their use as therapeutic targets (37). Furthermore, as the most abundant source of EVs in the circulatory system, pEVs are rich in molecules associated with regeneration and possess low immunogenicity and high delivery efficiency, demonstrating their potential as next-generation regenerative therapeutic tools (38). Studies have shown that PRP-EVs can attenuate apoptosis through activation of the protein kinase B (Akt)/Bcl-2-associated agonist of cell death (Bad)/B-cell lymphoma 2 (Bcl-2) signaling pathway, thereby contributing to bone protection in experimental models of osteonecrosis of the femoral head (39).

### 4.4 Current research challenges and prospects

#### 4.4.1 Common challenges in pEV research

With the continuous updates of the MISEV guidelines and the dissemination of consensus documents issued by several ISEV Task Forces, recent clinical studies have shown gradual improvements not only in EV isolation and characterization strategies, but also in terminology usage, reporting transparency, and functional validation practices (9, 13, 18, 31). For example, some studies have adopted size-exclusion chromatography (SEC), nanoparticle tracking analysis (NTA), and standardized flow cytometry workflows for pEV characterization, while others have incorporated the MIFlowCyt-EV framework to improve the reporting of flow cytometry parameters and gating strategies (13, 31). In addition, increasing attention has been paid to documenting EV origin, platelet activation conditions, and functional properties, reflecting growing awareness of the methodological principles advocated by ISEV.

Nevertheless, the implementation of these recommendations remains inconsistent across studies. Considerable heterogeneity persists in EV nomenclature, pre-analytical variables, isolation and characterization strategies, functional validation approaches, and reporting standards. Of particular concern is that differences in platelet activation status, EV generation conditions, and engineering modifications may substantially influence the composition and biological activity of pEVs. However, these factors are not consistently documented and are rarely incorporated into comparative analyses. As a result, direct comparisons between studies remain challenging, limiting the establishment of reference ranges, diagnostic thresholds, and clinical evaluation frameworks for pEVs.

Although the level of standardization in EV research has improved considerably in recent years, the challenges facing the field extend beyond technical standardization alone. Based on the current evidence, these common issues can be broadly categorized into two major aspects. The first is biological heterogeneity arising from differences in platelet activation status, EV generation conditions, and engineering modifications. The second is methodological heterogeneity resulting from variations in sample processing, isolation procedures, and characterization strategies. These two aspects are discussed separately below.

PEVs released from platelets activated through different receptor-mediated pathways exhibit distinct capacities to promote melanoma proliferation, invasion, and angiogenesis (52). This observation suggests that platelet activation status may be a key determinant of the functional heterogeneity of pEVs. The MISEV2023 guidelines recommend that operational classifications based on cellular origin or EV biogenesis conditions should be applied with caution (9). Therefore, rather than proposing a new classification system for EVs, we organized the current evidence according to the biological state of the parent platelets and the presence of engineering modifications. Specifically, pEVs reported in the literature can be broadly categorized into constitutively released pEVs derived from resting platelets, pEVs released from receptor-mediated activated platelets, and engineered pEVs generated through ex vivo modification strategies.

Recent studies have demonstrated marked differences in the immunomodulatory functions of constitutively released pEVs and those generated following receptor-mediated platelet activation (59). Furthermore, pEVs released after platelet activation by thrombin, collagen, or adenosine diphosphate (ADP) have all been associated with enhanced tumor cell migration, invasion, angiogenesis, and immune modulation (45, 52). Tavukcuoglu et al. directly compared pEVs generated through different platelet activation pathways, including collagen-related peptide (CRP), fucoidan, combined thrombin/collagen stimulation, and Ca²^+^ionophore stimulation. Their results revealed substantial differences in the ability of these pEVs to promote melanoma proliferation, invasion, angiogenesis, and transcriptomic remodeling (45). These findings provide direct evidence that platelet activation status contributes to the functional heterogeneity of pEVs. At the same time, engineered pEV-based platforms generated through ex vivo modification or biomimetic strategies have attracted increasing attention for drug delivery and cancer immunotherapy applications (52, 62, 64). For example, drug-loaded pEVs, platelet membrane-coated nanoparticles, and platelet-based immunomodulatory platforms have all demonstrated tumor-targeting potential in preclinical studies (52, 62, 64). Collectively, these findings suggest that platelet activation status and subsequent engineering modifications not only influence pEV release but may also affect their molecular cargo and biological functions.

Therefore, when integrating existing evidence, platelet activation conditions and engineering modifications should be incorporated as key contextual factors to improve the comparability and interpretability of findings across studies. Rather than establishing a new classification system for pEVs, we recommend that current research more systematically record platelet origin, activation methods, and engineering modifications, and combine these records with functional validation for comprehensive evaluation.

Beyond the limited clinical evidence, methodological heterogeneity remains a major factor limiting the reproducibility of current findings and the clinical translation of pEVs. The clinical studies included in this review exhibited substantial variations in sample collection, plasma preprocessing, pEV isolation procedures, and characterization strategies, which may significantly impact pEV counts, subpopulation composition, biological activity, and the resulting analyses (8, 9).

Among the included studies, centrifugation-based protocols were undoubtedly the most commonly employed approaches for plasma preprocessing and pEV isolation. Differential and ultracentrifugation formed the mainstream workflow, whereas alternative methods such as SEC, density gradient centrifugation, polymer-based precipitation, and immunoaffinity capture were applied less frequently (8, 9). We focused particularly on centrifugation-based approaches because they constitute the methodological foundation of most current clinical studies, thus exerting a decisive influence on the comparability and interpretation of published results.

Although centrifugation-based methods are widely adopted due to their relative simplicity, cost-effectiveness, and compatibility with routine plasma handling workflows, they are also a major source of methodological variability. Studies have shown that centrifugation speed, duration, rotor type, k-factor, braking conditions, and sequential centrifugation schemes can significantly affect the recovery efficiency, particle composition, and procoagulant activity of pEVs (67). Excessive centrifugal force may lead to loss or structural damage of small vesicles, whereas insufficient force can result in residual platelets, protein aggregates, and lipoprotein contamination. Moreover, many studies fail to fully report these critical parameters, making it difficult to reproduce and compare experimental protocols across different investigations.

Beyond centrifugation-based isolation methods, SEC has gained increasing attention in functional studies and proteomic analyses due to its ability to better preserve EV integrity while reducing contamination by soluble proteins (9, 33). However, its relatively low sample processing throughput limits broader application in large-scale clinical studies. Immunoaffinity-based approaches, which target platelet-specific markers such as CD41 and CD61, can enhance the specificity of pEV subtype enrichment. Nevertheless, these methods are relatively costly and may overlook biologically relevant heterogeneous EV subpopulations, thereby complicating standardization for clinical applications (9).In contrast, polymer-based precipitation methods are technically simple and generally achieve higher EV recovery rates, but co-precipitated non-EV contaminants can interfere with downstream functional assays and omics analyses (33). As a result, the use of these alternative approaches in current clinical pEV research remains relatively limited.

Regarding pEV characterization, the studies included in this review indicate that flow cytometry remains the most commonly employed analytical technique, primarily used for quantifying circulating pEVs and profiling their phenotypes. Nevertheless, the sensitivity of flow cytometry for detecting small EVs remains limited, potentially leading to underestimation of certain small pEV subpopulations. In addition, substantial heterogeneity exists among studies in gating strategies, fluorescence thresholds, calibration beads, and data reporting units, further amplifying interstudy variability (26).

To improve the reliability of pEV characterization, other commonly used complementary techniques in the included studies include NTA, transmission electron microscopy (TEM), Western blot, and proteomic profiling (8, 9, 20). Specifically, NTA is primarily applied to assess particle concentration and size distribution, TEM is used to observe vesicle morphology, and Western blot and proteomics are employed to evaluate marker expression and molecular composition. However, the clinical application of these techniques remains constrained by factors such as analysis throughput, sample quality, experimental standardization, and lipoprotein contamination (9, 68). Consequently, a unified framework for clinical pEV characterization has not yet been established.

#### 4.4.2 Research bottlenecks unique to hot fields and coping strategies

Despite growing evidence supporting important roles for pEVs in tumor progression and therapeutic applications, several tumor-specific challenges remain unresolved.

First, the biological effects of pEVs in cancer appear to be highly context-dependent and may exert both tumor-promoting and tumor-suppressive functions. Most studies have focused on the ability of pEVs to enhance tumor cell proliferation, invasion, angiogenesis, and therapeutic resistance (41–54). However, recent evidence suggests that pEVs may also enhance antitumor immune responses under certain conditions, for example by transferring integrin β3 to tumor-infiltrating T cells (51). These findings indicate that the functional consequences of pEVs may depend on multiple factors, including platelet activation status, cargo composition, recipient cell type, and characteristics of the tumor microenvironment (59). Future studies should directly compare pEVs generated under different platelet activation conditions and systematically characterize their cargo profiles using multi-omics approaches. Combining single-vesicle analysis with functional validation may help identify the molecular determinants responsible for the divergent effects of pEVs in tumor progression and antitumor immunity.

Second, the application of pEVs as liquid biopsy biomarkers is still limited by insufficient specificity. Multiple studies have demonstrated that circulating pEV levels and their molecular cargo are closely associated with tumor progression, prognosis, and treatment response (44, 50, 55–58). However, platelet activation is not unique to cancer and can also be triggered by infection, inflammation, surgical trauma, and hypercoagulable conditions. Consequently, elevated circulating pEV levels do not necessarily reflect tumor burden directly. Future prospective studies should further standardize sample collection and processing procedures and incorporate platelet activation markers, such as soluble P-selectin and platelet factor 4 (PF4), into multivariable analytical models. Such approaches may help reduce the confounding effects of nonspecific platelet activation and improve the specificity and clinical utility of pEVs as cancer biomarkers.

Finally, although engineered pEV-based platforms have shown considerable promise for drug delivery and cancer immunotherapy (38, 52, 60–64), their long-term safety remains insufficiently characterized. Native pEVs are intrinsically involved in angiogenesis, immune regulation, and remodeling of the tumor microenvironment (45, 52–54). Therefore, whether engineering modifications can completely eliminate their potential tumor-promoting effects remains unclear. In addition, the immunogenicity, biodistribution, and interactions with the coagulation system following repeated administration have not been systematically evaluated. Future studies should place equal emphasis on therapeutic efficacy and safety assessment. In particular, standardized preclinical evaluation frameworks should be established to systematically investigate the biodistribution, immunogenicity, and procoagulant potential of engineered pEVs following repeated administration. In addition, comparative studies between native and engineered pEVs are needed to determine whether engineering modifications alter their intrinsic biological activities, including their effects on angiogenesis, immune regulation, and tumor progression.

Despite the considerable potential of pEVs in regenerative medicine, several key challenges continue to limit their further application. First, biological heterogeneity remains a major obstacle, potentially affecting the reproducibility and comparability of current studies (9, 65). PEVs derived from different sources or subpopulations may exhibit substantial differences in pro-angiogenic activity, immunomodulatory capacity, and tissue repair potential, thereby constraining the consistency of existing research findings. Second, the regenerative effects of pEVs are highly dependent on the pathological microenvironment. Under complex conditions such as diabetes, chronic inflammation, or ischemia-hypoxia, local oxidative stress, inflammatory mediators, and metabolic alterations can influence the stability of pEVs, their cellular uptake efficiency, and downstream signaling activation, ultimately affecting therapeutic outcomes (69). Moreover, although pEVs are generally considered to have relatively low immunogenicity, they naturally carry certain platelet-derived procoagulant components, which may pose potential thrombotic risks and off-target effects under systemic administration (6, 65). Notably, studies have shown that in specific pathological contexts, such as sepsis, pEVs may exacerbate inflammation and disease progression, further highlighting their context-dependent and bidirectional biological effects (65). Importantly, current evidence indicates that EVs in the circulation are rapidly cleared by the mononuclear phagocyte system, particularly in the liver and spleen, resulting in limited *in vivo* retention and reduced bioavailability, which may further constrain the sustained efficacy and targeted delivery potential of pEVs (70).

In summary, the role of pEVs in regenerative medicine has been supported by accumulating experimental and preclinical evidence. However, future research should move beyond simply validating their beneficial mechanisms and instead focus on achieving more precise and controllable therapeutic applications. For example, intelligent delivery systems that respond to the injured microenvironment could be developed, or engineered pEVs with surface-targeting modifications could be constructed to enable site-specific delivery. Such approaches may improve local retention while reducing the potential safety risks associated with systemic administration (71). In addition, future studies may integrate proteomics, lipidomics, and single-particle characterization technologies to identify key functional molecules involved in tissue repair under different pathological conditions. This would facilitate more precise functional engineering of pEVs. Furthermore, molecular components that may contribute to excessive inflammation or procoagulant activity should be systematically identified and regulated to improve the safety and controllability of pEV-based therapeutic strategies.

## 5 Limitations

This study has several limitations. First, only the WoSCC and PubMed databases were included, which may not fully capture all relevant publications and could introduce publication bias. For example, the coverage of Chinese-language journals in WoSCC is relatively limited, potentially leading to an underestimation of the contributions and influence of Chinese researchers and institutions. Second, the clinical analysis primarily focused on identifying major translational trends and research directions and did not further explore the interactions among different clinical themes or their longitudinal evolution over time. Third, the terminology used in the pEV field has evolved substantially over time. Early studies frequently employed terms such as”exosomes, ““microparticles, “and “microvesicles, “whereas more recent studies increasingly adopt the nomenclature recommended by the MISEV guidelines, including sEVs and lEVs. Such inconsistencies in terminology may have influenced both the LDA-based topic classification and the interpretation of bibliometric trends. In addition, LDA topic modeling is an unsupervised machine-learning approach, and the resulting topic structure may be affected by factors such as corpus size, the predefined number of topics, and patterns of terminology usage. Although topic assignments were validated using complementary approaches, including keyword burst analysis, semantic overlap and ambiguous boundaries between certain topics may still exist.

Furthermore, author rankings based on citation metrics should be interpreted with caution. Some highly cited authors have not only contributed a substantial body of original research but have also participated in the development and revision of international consensus guidelines, such as MISEV. Because these guidelines are widely cited across the extracellular vesicle field, their citation counts may substantially exceed those of conventional research articles, thereby amplifying the bibliometric rankings of the associated authors. Consequently, citation-based indicators may not fully reflect an individual author’s direct contributions to pEV research.

Despite these limitations, the integration of LDA topic modeling with clinical evidence provides complementary perspectives that strengthen the overall reliability of the findings. This combined approach offers a comprehensive overview of the evolving research landscape and may serve as a useful reference for future investigations and clinical translation in the pEV field.

## 6 Conclusion

This study employed a combined approach of bibliometrics and LDA topic modeling to map the knowledge landscape and developmental trajectory of the pEVs field. From 2015 to 2025, research on pEVs has continued to grow, with the focus shifting from the exploration of pathological mechanisms to cutting-edge areas such as tumor research, regenerative medicine, and drug delivery. Current research hotspots in the field center on the molecular mechanisms of pEVs in tumor microenvironment remodeling, immune regulation, and tissue repair, as well as the standardization of separation and detection techniques for EVs as biomarkers. However, tseveral challenges continue to hinder the clinical implementation of pEVs. Methodological heterogeneity remains substantial across studies, particularly regarding platelet activation conditions, EV isolation procedures, characterization strategies, and reporting standards, which limits interstudy comparability and reproducibility. In addition, most clinical investigations remain exploratory and are based on relatively small cohorts, whereas the therapeutic applications of pEVs in oncology and regenerative medicine are still largely confined to preclinical research. In summary, pEV research is at a critical juncture of transition from basic research to clinical application, possessing immense clinical potential. Overall, pEV research is entering a transitional stage from mechanistic exploration toward clinical translation. Future progress will require greater methodological standardization, rigorous adherence to MISEV recommendations, improved quality-control frameworks, and well-designed large-scale clinical studies to fully evaluate the diagnostic, prognostic, and therapeutic potential of pEVs.

## Data Availability

The original contributions presented in the study are included in the article/**Supplementary Material**. Further inquiries can be directed to the corresponding author.
