# Supplementary figures and images for: The research landscape and evolutionary trends of platelet-derived extracellular vesicles: a bibliometric and LDA analysis (2015–2026)
Source: Front Oncol. 2026 Jul 8;16:1854626. doi: 10.3389/fonc.2026.1854626 (PMC13388083; doi:10.3389/fonc.2026.1854626)

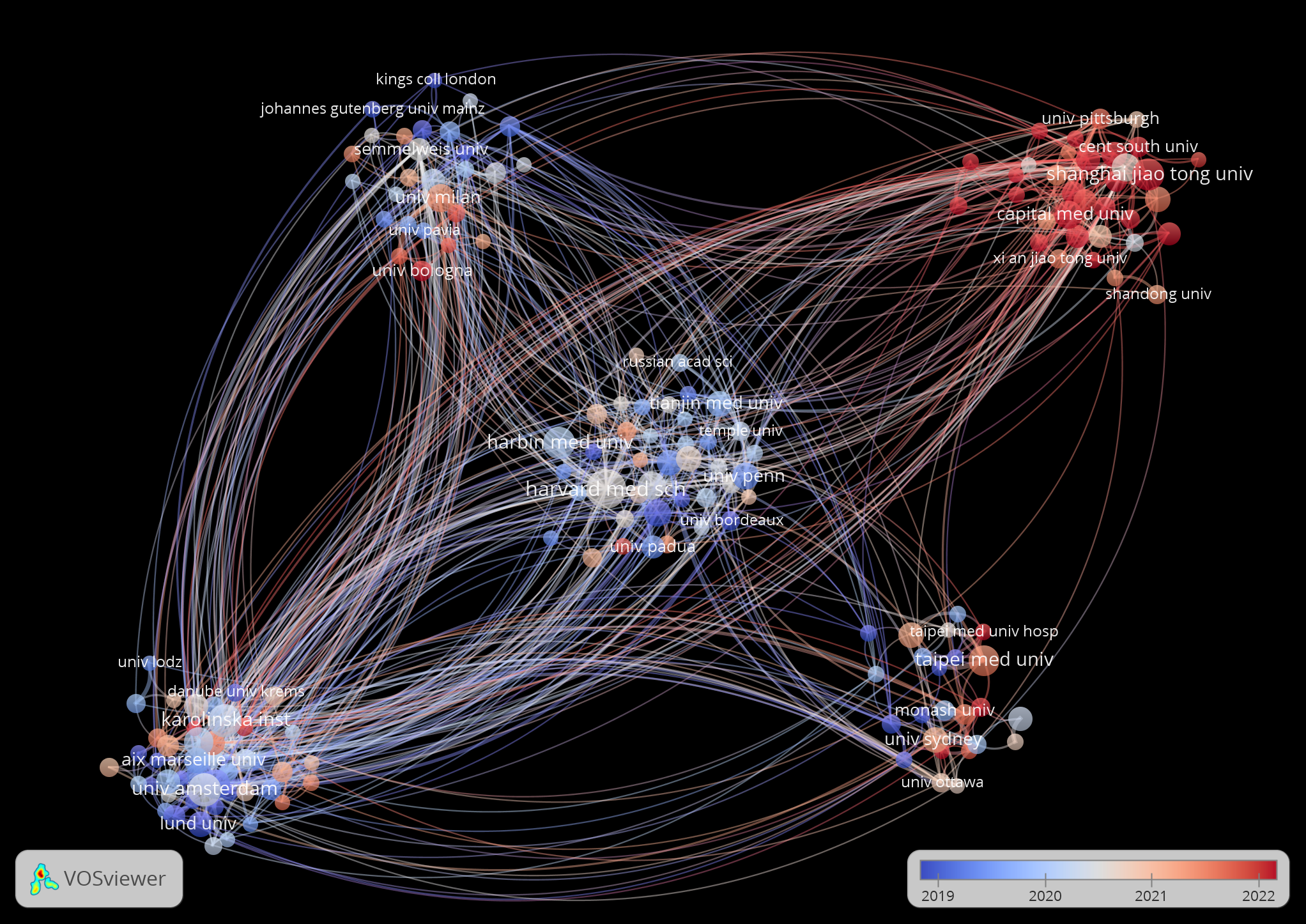

Supplement: Supplementary file 3 [file Image1.tif]
