# Supplementary material for: The research landscape and evolutionary trends of platelet-derived extracellular vesicles: a bibliometric and LDA analysis (2015–2026)
Source: Front Oncol. 2026 Jul 8;16:1854626. doi: 10.3389/fonc.2026.1854626 (PMC13388083; doi:10.3389/fonc.2026.1854626)
Supplement: Supplementary file 6 [file Table3.docx]

Inclusion criteria

1. The study subjects are platelet-derived extracellular vesicles( Includes synonymous expressions ), and pEVs are the primary or secondary endpoints of the study.

2. The study includes complete clinical trial data from human subjects.

3. The full text of the literature is available, and key research data is complete and can be extracted in a standardized manner.

Exclusion criteria

1. The study focuses on non-platelet-derived vesicles (from endothelial cells, red blood cells, white blood cells, etc.).

2. The study lacks human clinical trial data and is based solely on in vitro and animal studies. It lacks key clinical data or contains instances of duplicate publication.

3. The study merely reaffirms established conclusions in the field and does not yield any new clinical findings.

4. Low relevance to the topic: Does not address extracellular vesicles, or is not relevant to the clinical or translational aspects of pEVs.
