# Supplementary material for: The research landscape and evolutionary trends of platelet-derived extracellular vesicles: a bibliometric and LDA analysis (2015–2026)
Source: Front Oncol. 2026 Jul 8;16:1854626. doi: 10.3389/fonc.2026.1854626 (PMC13388083; doi:10.3389/fonc.2026.1854626)
Supplement: Supplementary file 7 [file Table4.docx]

**PubMed database search formula**

((( "Platelet*"[Title/Abstract] OR "Thrombocyte*"[Title/Abstract]) AND ("Extracellular Vesicle*"[Title/Abstract] OR "Apoptotic Bod*"[Title/Abstract] OR "Exovesicles*"[Title/Abstract] OR "Exosome*"[Title/Abstract] OR "Ectosome*"[Title/Abstract] OR "Microvesicle*"[Title/Abstract] OR "Oncosome*"[Title/Abstract] OR "Microparticle*"[Title/Abstract] OR "Endosome*"[Title/Abstract])) AND "English"[lang]) AND (("2015/01/01"[Date - Publication] : "2026/02/28"[Date - Publication])) Filters: Clinical Trial
